# Low-dose hCG as trigger day and 35 hr later have different ovarian hyperstimulation syndrome occurrence in females undergoing In vitro fertilization: An RCT

**Published:** 2017-11

**Authors:** Marzieh Aghahosseini, Ashraf Aleyasin, Venus Chegini, Victoria Chegini

**Affiliations:** 1 *Department of Infertility, Tehran University of Medical Sciences, Tehran, Iran.*; 2 *Department of Obstetrics and Gynecology, Qazvin University of Medical Sciences, Qazvin, Iran.*; 3 *Qazvin University of Medical Sciences, Qazvin, Iran.*

**Keywords:** Ovarian hyperstimulation syndrome, Infertility, Assisted reproductive technology, In vitro fertilization, Human chorionic gonadotropin

## Abstract

**Background:**

Ovarian hyperstimulation syndrome (OHSS) is an iatrogenic complication, which can cause high morbidity and mortality. Use of gonadotropin releasing hormone (GnRH) agonist instead of human chorionic gonadotropin (hCG) in GnRH antagonist cycles causes luteinizing hormone surge by GnRH stimulation which reduces the risk of OHSS by reducing the total amount of gonadotropin; however, there is no possibility of transferring fresh embryos.

**Objective:**

The current study aimed to evaluate the effect of hCG along with GnRH agonist administration in the occurrence of OHSS and pregnancy rate in females undergoing in vitro fertilization.

**Materials and Methods:**

The current randomized clinical trial was conducted on 80 cases in 2 groups. Gonal-F was used to stimulate the oocyte from the second day of menstruation. When follicle size was 12-14 mm, GnRH antagonist was added to the protocol till the detection of more than two follicles greater than 18 mm. Then, GnRH agonist was added to the protocol as a trigger. In group A, 35 hr after the administration of GnRH agonist, the low-dose human hCG, 1500 IU, was used. In group B, low-dose hCG, 1500 IU, was used at the same time by GnRH agonist administration. The rate of pregnancy, OHSS, and its severity were compared between 2 groups within 2 wk.

**Results:**

There was no significant difference regarding chemical and clinical pregnancies between the 2 groups. Severe OHSS was significantly higher in group B (p= 0.03).

**Conclusion:**

Administration of hCG 35 hr after GnRH agonist administration results in lower rate of severe OHSS.

## Introduction

Infertility is defined as the inability to conceive after one year of unprotected sex that affects one-fifth of couples (1). Along with the increase of infertile couples, the number of assisted reproductive technology (ART) cycles have increased. One of the most important parts of in vitro fertilization (IVF) cycles is ovarian stimulation, which leads to multiple follicle developments (2). 

It provides an increased number of retrieved oocytes and provides the opportunity to select the best quality embryos (3). Although this strategy improves the ART outcome, it may lead to iatrogenic occurrence of ovarian hyperstimulation syndrome (OHSS) (4). OHSS is associated with mortality, morbidity, and ART cycle cancelation in fresh embryo cycles. The moderate and severe forms of OHSS could occur in 3-10% of all ART cycles; in high-risk females, it could reach 20% (5-9). Factors such as polycystic ovarian syndrome (PCOS), high FSH dosage, high ratio between luteinizing hormone (LH)/ FSH, high human chorionic gonadotrophin (hCG) dosage, high inhibin A and B levels, high vascular endothelial growth factor, and other interleukins levels, and decreased alpha-2-macroglobulin levels are considered as OHSS risk factors (5, 10-14).

Previously, hCG was used as the trigger in ART cycles, but it was shown that its luteotropic activity leads to OHSS as its mechanism is similar to LH with a longer half-life (15). In subjects at higher risk of developing OHSS, application of gonadotropin-releasing hormone (GnRH) antagonist with GnRH agonist trigger are introduced for oocyte maturation (16). Simultaneous application of low-dose hCG (1500 IU) with GnRH agonist or injection after 36 hr could be useful to support luteal phase (17). Shapiro and colleagues reported excellent reproductive outcome after administration of hCG along with GnRH agonist as the trigger (18). On the other hand, Humaidan and colleagues found that administration of hCG 35 hr after GnRH agonist administration could rescue the corpus luteum (19).

As there are few studies on this issue, the current study aimed to evaluate the effect of hCG along with GnRH agonist administration in the occurrence of OHSS and pregnancy rate in females undergoing IVF.

## Materials and methods

The current randomized, clinical trial was conducted in Omid-e-Tehran Infertility Clinic from March 2014 to March 2015. All subjects diagnosed with PCOS and had OHSS risk factors and were in a high risk of this syndrome. 

The inclusion criteria were maternal age 18-39 yr, normal FSH level, history of PCOS due to Rotterdam criteria (7), number of antral follicles more than 20, anti-Mullerian hormone level >3.5 ng/mL, number of follicles >18 mm more than 20, and estradiol level more than 4000 pg/mL. Patients were blindly divided into group A and group B through the simple random sampling method blindly by a technician (by the computer-generated randomization). IVF protocol was GnRH antagonist protocol.

Gonal-F (75 uBD) was used for oocyte stimulation from the second day of menstruation. When follicle size was 12-14 mm, GnRH antagonist (Cetrotide 0.25 mg) was added to the protocol till the detection of more than two follicles greater than 18 mm. Then, GnRH agonist (buserelin 0.5 mg) was added to the protocol as the trigger. In group A, 35 hr after GnRH agonist administration, low-dose human chorionic gonadotropin (hCG, corogan), 1500 IU, was used. In group B, low-dose hCG, 1500 U, was used at the same time by GnRH agonist administration. In both groups, 34-36 hr after the administration of GnRH agonist, under transvaginal sonographic guidance, oocytes were picked-up. OHSS and its severity were compared between the 2 groups within 2 wk. Mild and moderate OHSS were considered by increased weight, abdominal discomfort and/or distension, nausea, vomiting, and/or ascites (5). 

Severe OHSS is a kind of disease that in addition to the above symptoms is associated with hypovolemia, hemoconcentration, coagulopathies electrolyte imbalance, respiratory and liver failure, increased risk of thromboembolism, and decreased kidney function, which included the exclusion criteria (5, 6).In group B, embryo transfer was done in 27 out of 40 subjects, and embryo transfer was canceled in 13 patients with severe OHSS. In group A, embryo transfer was done in 35 out of 40 subjects, and embryo transfer was canceled in 5 patients with severe OHSS. The decision to transfer the embryo was made on the basis of the patients’ symptoms. Chemical pregnancy was determined by the detection of serum β-hCG 2 wk after embryo transfer. Clinical pregnancy was identified by the development of a gestational sac (20).

**Ethical consideration**

All patients were asked to sign the informed consent forms before the procedure. The study was approved by Ethics Committee of Tehran University of Medical Sciences (IR.TUMS.REC.1394.1508).

**Statistical analysis**

The sample size was calculated based on Radesic and Tremellen study, which required at least 27 samples per group (20). SPSS (Statistical Package for the Social Sciences, version 22.0, SPSS Inc, Chicago, Illinois, USA) was used for data analysis. Normality of data was evaluated using Kolmogorov-Smirnov tests. The Student t-test and the Fisher exact tests were used to compare continuous and categorical variables. A p-<0.05 was considered statistically significant.

## Results

**Figure 1:**
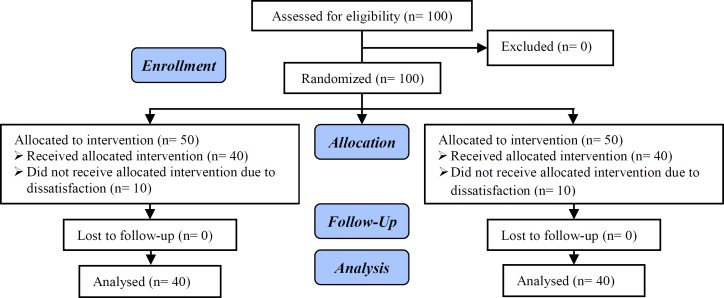
Study flow chart.

One hundred females were enrolled in the current trial. Ten subjects in each group withdrew due to dissatisfaction. Finally, forty patients in each group finished the study (Figure 1). Demographic characteristics of the study groups has been abbreviated on Table I. There was no significant difference regarding chemical and clinical pregnancies in the two groups (Table II). Severe OHSS was significantly higher in group B. 

**Table I:** Demographic characteristics of the study groups

**p-value**	**Group B**	**Group A**		
0.8	30 ± 3.9	29.8 ± 4.3	Age*	
0.3	4.3 ± 2	4.8 ± 2.9	Duration of infertility*	
Type of infertility**				
0.7	34 (85%)	35 (87.5%)	Primary	
	6 (15%)	5 (12.5%)	Secondary	
0.2	25.2 ± 2.9	26.2 ± 2.9	BMI*	
History of OHSS**				
0.4	10 (25%)	8 (20%)	Yes	
	30 (75%)	32 (80%)	No	

BMI: body mass index

OHSS: Ovarian hyperstimulation syndrome

The Student t-test and the Fisher exact tests were used.

* Data presented as mean±SD.

** Data presented as n (%)

**Table II:** Laboratory findings in the study groups

**p-value**	**Group B**	**Group A**		
0.2	26.5 ± 8.9	24.4 ± 8.5	Retrieved oocytes*	
0.1	16.1 ± 6.6	14.2 ± 6.4	Oocytes in metaphase II*	
Chemical pregnancy**				
1	19 (47.5%)	19 (47.5%)	Yes	
	21 (52.5%)	21 (52.5%)	No	
Clinical pregnancy**				
0.3	18 (45%)	19 (47.5%)	Yes	
	22 (55%)	21 (52.5%)	No	
OHSS**				
0.03	27 (67.5%)	35 (87.5%)	No or mild	
	13 (32.5%)	5 (12.5%)	Severe	

The Student t-test and the Fisher exact tests were used.

OHSS: Ovarian hyperstimulation syndrome.

* Data presented as mean±SD.

** Data presented as n (%)

## Discussion

OHSS is an iatrogenic complication in ART cycles, which can cause high morbidity and mortality. Recent studies showed that using GnRH agonist instead of hCG in GnRH antagonist cycles, causes LH surge by GnRH stimulation and can reduce the risk of OHSS by reducing the total amount of gonadotropin; however, there is no possibility of transferring fresh embryos. The current study tried to compare the total of OHSS and pregnancy using low-dose hCG (1500 U) immediately or 35 hr after GnRH agonist considering the initial goal in infertility treatment that is transferring the fresh embryo and improving luteal phase without increasing the risk of OHSS.

The results of the current study showed no significant difference regarding the chemical and clinical pregnancies in group A and group B, while sever OHSS rate was significantly higher in group B. Seyhan and colleagues evaluated 23 infertile females treated with GnRH agonist trigger + hCG support protocol in a GnRH antagonist. They found 6 (26%) subjects who developed severe OHSS, while this rate was 5 (12.5%) in the current study in Group A (21). 

Radesic and Tremellen assessed 71 females who received GnRH agonist trigger + hCG support protocol as IVF protocol. They included females with more than 13 follicles of ≥12 mm on the day 8 or 9 of the stimulation cycle. Oocyte maturation was done by 1500 IU hCG administration 37 hr after leuprolide acetate use. Vaginal progesterone and oral estradiol valerate were used for luteal phase support. Chemical pregnancy rate was 60% and the clinical pregnancy rate was 52%. Only 1 case developed severe OHSS (20). Nargund and colleagues reported no moderate or severe OHSS in females receiving a minimum dose of hCG (2500 IU) at the same time by the trigger (22).

Humaidan and colleagues allocated females at higher risk of OHSS (follicles ≥11 mm diameter; 15-25 numbers) into 2 groups: ovulation triggering with a bolus of GnRH agonist followed by a single dose of 1500 IU hCG after 35 hr in group A, and receiving 5000 IU hCG by the trigger in group B. Clinical pregnancy rate was 35% in group A and 29% in group B. OHSS rate was 0% in group A and 3.4% in group B (23). They also divided the low-risk group of OHSS (follicles ≥11 mm diameter; less than 15 numbers) into 2 groups: group C received a bolus of 0.5 mg GnRH agonist followed by a bolus of 1500 IU hCG on the day of oocyte retrieval and 1500 IU hCG after oocyte retrieval, while group D received 5000 IU hCG at the same time by the trigger. Pregnancy rate was 34% in group C and 28% in group D (p=0.2). 

OHSS was detected in 1.6% of group C subjects and none in group D (23). In a systematic review conducted by Yousef and colleagues, 11 RCTs with 1055 cycles were evaluated. The authors concluded that GnRH agonists should not be routinely used as a final oocyte maturation trigger as pregnancy rate was lower in the subjects under the treatment of GnRH agonists. They suggested that this protocol was useful for the females at higher risk of OHSS (24). The current study showed that the rate of OHSS was significantly lower in group A than group B. Direct and indirect effects of hCG on endometrial cavity were reported previously (20). 

It is suggested that hCG boosts the production of corpus luteum hormones such as relaxing with positively helped endometrial development (25). A bolus dose of hCG is used for the endogenous LH surge to induce ending of the oocyte maturation (23). The half-life of hCG is significantly longer than that of the endogenous LH, which leads to prolonged luteotropic effect (26, 27). 

Previously, administration of GnRH agonist alone as a trigger in IVF/ICSI cycles standard as the luteal phase support was associated with higher pregnancy loss rate (28, 29). Adding hCG as luteal phase support on the day of oocyte retrieval causes pregnancy loss reduction (19, 30). In a systematic review conducted in 2015, simultaneous application of GnRH agonist with 1500 U hCG was considered as a good strategy to increase pregnancy rate, while associated with higher rate of OHSS. The application of hCG was associated with higher miscarriage (31). There was no significant difference for the chemical and clinical pregnancy rates between two groups. 

It is recommended using GnRH agonist as a trigger in GnRH antagonist cycles; in addition, to support luteal phase, low-dose hCG (1500 IU) should be administered to transfer the fresh embryo.

## Conclusion

According to the results of the current study, following administration of 1500 IU hCG in GnRH antagonist cycles on the day of oocyte retrieval, the rate of severe OHSS significantly reduced, compared to administration of hCG 1500 IU + GnRH agonist at the same time, but there were no changes in the number of oocytes in metaphase II and rate of pregnancy.

## Note

***Registration ID in IRCT: IRCT2016031427045N1***
